# Real-world validity of randomized controlled phase III trials in newly diagnosed glioblastoma: to whom do the results of the trials apply?

**DOI:** 10.1093/noajnl/vdab008

**Published:** 2021-02-26

**Authors:** Erlend Skaga, Marthe Andrea Skretteberg, Tom Børge Johannesen, Petter Brandal, Einar O Vik-Mo, Eirik Helseth, Iver A Langmoen

**Affiliations:** 1 Vilhelm Magnus Laboratory for Neurosurgical Research, Institute for Surgical Research and Department of Neurosurgery, Oslo University Hospital, Oslo, Norway; 2 Institute of Clinical Medicine, Faculty of Medicine, University of Oslo, Oslo, Norway; 3 Department of Registration, Cancer Registry of Norway, Oslo, Norway; 4 Department of Oncology, Oslo University Hospital, Oslo, Norway

**Keywords:** clinical trials, eligibility, glioblastoma, selection bias, survival

## Abstract

**Background:**

The survival rates in population-based series of glioblastoma (GBM) differ substantially from those reported in clinical trials. This discrepancy may be attributed to that patients recruited to trials tend to be younger with better performance status. However, the proportion and characteristics of the patients in a population considered either eligible or ineligible for trials is unknown. The generalizability of trial results is therefore also uncertain.

**Methods:**

Using the Cancer Registry of Norway and the Brain Tumor Database at Oslo University Hospital, we tracked all patients within a well-defined geographical area with newly diagnosed GBM during the years 2012–2017. Based on data from these registries and the medical records, the patients were evaluated for trial eligibility according to criteria employed in recent phase III trials for GBM.

**Results:**

We identified 512 patients. The median survival was 11.7 months. When we selected a potential trial population at the start of concurrent chemoradiotherapy (radiotherapy [RT]/ temozolomide [TMZ]) by the parameters age (18–70 y), passed surgery for a supratentorial GBM, Eastern Cooperative Oncology Group (ECOG) ≤2, normal hematologic, hepatic and renal function, and lack of severe comorbidity, 57% of the patients were excluded. Further filtering the patients who progressed during RT/TMZ and never completed RT/TMZ resulted in exclusion of 59% and 63% of the patients, respectively. The survival of patients potentially eligible for trials was significantly higher than of the patients not fulfilling trial eligibility criteria (*P* < .0001).

**Conclusions:**

Patients considered eligible for phase III clinical trials represent a highly selected minority of patients in a real-world GBM population.

Key Points

1. A minority of GBM patients are eligible for clinical trials.
2. Patients in trials are not representative for a real-world GBM population.
3. The extrapolation of trial results to the real-world population is uncertain.

**Importance of the Study**

Clinical trials are fundamental for therapeutic advances in GBM and according to the National Comprehensive Cancer Network, the best management of a cancer patient is in a trial. However, by current trial inclusion and exclusion criteria, only a selected group of patients can be considered eligible for trials. This bias not only restricts patients from receiving the best care, but also makes it difficult to extrapolate trial results to a real-world population. Here, we estimated the proportion of GBM patients who did not fulfill eligibility criteria for trial participation and compared the characteristics of patients considered trial participants to those excluded. We found that approximately 60% of patients were ineligible for trials. These patients were older, had worse performance status, received less treatment and had worse survival. This implies that the current trial landscape inadequately reflects the population, and that generalizability of trials results into clinical practice carries considerable uncertainty.

The median survival of patients with newly diagnosed glioblastoma (GBM), the most frequent and most malignant primary brain tumor, is commonly claimed to be about 15 months.^1^ Although valid for the subgroup of patients who are entered into randomized clinical trials (RCT) and typically undergo multimodal treatment with surgery, radiotherapy (RT) and chemotherapy with temozolomide (TMZ), this survival rate contrasts observations made in unselected populations of real-world GBM patients, where the survival is reported to be considerably shorter.^2–5^

The better outcome of patients in clinical trials is attributed to the selected population of patients being studied. From a heterogeneous patient population, trials infer a selection bias and recruit a more homogenous group of a well-defined population of patients. This selection aims to minimize confounders that might affect trial outcome.^6^ Compared to real-world patients, phase III trials in GBM usually enroll patients who are younger, have a more favorable performance status, and are more likely to have undergone tumor resection surgery,^7–16^ all of which are established as strong prognostic factors that influence survival.^17–19^ As a consequence, patients in trials have better outcomes than can be expected in a population setting, where inclusion and exclusion criteria do not exist and follow-up might be less rigorous.^20^

There is, however, a lack of data describing the proportion and characteristics of the patients who do not fulfill standard eligibility criteria for phase III clinical trials in GBM. The generalizability of trial results is also therefore uncertain. Due to several prospectively maintained national and regional databases that can be linked, combined with a single-payer universal health care system, Norway has a unique opportunity for population-based studies. Here, we utilized data from the Cancer Registry of Norway and the Brain Tumor Database at the Oslo University Hospital to evaluate the proportion and characteristics of real-world patients with newly diagnosed GBM that fulfill or violate eligibility criteria in phase III trials.

## Materials and Methods

### Population Base

Each person in Norway is registered in The National Population Register with a unique ID number and contact information that facilitates contact between health care officials and individual patients. In this study, we collected data from the counties Akershus, Buskerud, Oslo, Telemark, Vestfold, and Østfold since all patients from these counties receive their oncological treatment at Oslo University Hospital (OUH). This defined geographical area comprised 2.2 million people (43.9% of the Norwegian population) during the study period.

### Cancer Registries

To identify the patients in this study, we used the Cancer Registry of Norway (CRN) and the Brain Tumor Database (BTD) at Oslo University Hospital.

The CRN was founded in 1951, and it maintains a prospective database of all tumors diagnosed in Norway, including both malignant and benign tumors of the central nervous system (CNS). Reporting to the registry is compulsory by law, and the registry is based on clinicians’ reports, pathology reports, and information from death certificates reporting neoplastic disease. The quality of the registry is maintained by ensuring that missing reports from attending clinicians or pathologists are requested by direct contact. Data from the CRN have undergone quality control and are valid for population studies.^21^ The tumors from the time period in this study were coded according to the third revision of the International Classification of Diseases for Oncology (ICD-O-3, primary GBM, site codes 710–719, and histology codes 9440–9442). The registry also retrieves data electronically, ensuring information on oncological treatment (RT and TMZ) in all patients. The unique identification number of each individual is maintained by the government and change to death status is conveyed to the CRN. This study has used data from the CRN. The interpretation and reporting of these data are the sole responsibility of the authors, and no endorsement by the CRN is intended nor should be inferred.

The BTD is a prospective database containing details of all tumor resections and biopsies carried out in the Department of Neurosurgery, OUH. The registry retrieves data electronically from every patient, including name, unique ID number, sex, age at diagnosis, tumor localization, type of surgical treatment, pathology, and survival.

### Patient Data

Using the BTD we identified all patients diagnosed with GBM (*n* = 512) between 2012 and 2017 from the defined counties. By using the CRN, we could also identify the patients who were diagnosed by MRI only. Two of the authors independently undertook a systematic review of the medical records of the individual patients in order to obtain the required data which included the following; sex, age at diagnosis, tumor localization (dichotomized into supra- or infratentorial), performance status by the Eastern Cooperative Oncology Group (ECOG) evaluated preoperatively, as well as before the start, at midway and at the end of concurrent RT/TMZ, type of primary surgery (no surgery, stereotactic biopsy, open biopsy or resection), primary oncological treatment (RT and TMZ), other comorbidities (heart, hepatic, immunologic, lung, psychiatric, or renal disease, coagulation insufficiency, previous malignancy, previous oncological treatment, and other disorders of the CNS, eg, dementia), medication use before the diagnosis of GBM (number of drugs taken daily that required prescription from a physician), the use and doses of steroids before, midway and at the end of concurrent RT/TMZ, disease status (progressive or not progressive disease) until finished radiochemotherapy phase, and blood levels of white blood cells (WBC), thrombocytes (Tbc), creatinine, bilirubin, aspartate amino transferase (AST), and alanine amino transferase (ALT). The time of death is recorded in both the CRN and BTD as obtained from the Norwegian Population Register. The last date of follow-up was July 6, 2020.

### Definition of Variables

We used the phase III trials in newly diagnosed GBM that were published over the last 10 years as reference trials.^7–16^ We assessed the parameters used for inclusion and exclusion of patients in the respective trials (reviewed in Supplementary Table S1) and derived a list of the most commonly and uniformly used criteria that a patient must fulfill to be enrolled in a phase III trial for newly diagnosed GBM. These variables concern patient age, comorbidities, performance status, physiologic parameters (hematologic, hepatic, renal function), tumor localization, pathology, oncological treatment, and glucocorticoid use, as elaborated below. Based on the time of randomization used in different trials (Supplementary Table S1), we defined 3 time points for potential study recruitment: (1) before concurrent RT/TMZ, (2) midway through concurrent RT/TMZ, and (3) at the end of the concurrent RT/TMZ.

We used the ECOG scale to classify patient performance status as this is the scale used prospectively by physicians at OUH. In cases where the ECOG status was not specified in the medical records, it was assessed retrospectively by 2 independent researchers. Tumors that extended both supra- and infratentorial were categorized as infratentorial. The surgical procedure (stereotactic biopsy, open biopsy, and resection) was categorized according to the procedural description by the neurosurgeon. We did not distinguish between subtotal and total resections. Adjuvant treatment was considered completed in patients who received a radiation dose of ≥54 Gy and ≥5 weeks of concurrent TMZ (75 mg/m^2^). Patients were categorized as having received a suboptimal dose of RT if they received <54 Gy. This group included older patients who received hypofractionated treatment (3 Gy × 10 or 2.67 Gy × 15) and patients where RT was stopped because of side effects, tumor progression, or death. Patients were categorized as having discontinued TMZ treatment if TMZ was withdrawn before the completion of the fifth week (out of 6 weeks) of the concurrent phase due to side effects, toxicity, progression, or death.

We defined threshold levels of hematologic, hepatic, and renal function according to levels commonly used in published phase III trials in GBM.^7–16^ Patients were excluded if they had WBC < 1.5 ×10^9^/L, tbc < 100 × 10^9^/L, creatinine >150 µmol/L, bilirubin >34 µmol/L or AST/ALT >3 times above the upper reference limit of the hospital laboratory at the defined time points. For disqualifying comorbidities, we used the diseases commonly listed as reasons for trial exclusion according to published phase III trials.^7–16^ This included patients with active heart disease (NYHA 3–4, unstable angina pectoris, myocardial infarction within last 6 months), active hepatic disease (hepatic insufficiency), or active lung disease (COPD requiring hospitalization within the last 6 months), patients with bleeding disorders, immunologic disorders with inherited or acquired immunodeficiency or iatrogenic immunosuppression other than glucocorticoid use due to GBM, recent (within the last 6 months) intracranial bleeding, recent (within the last 6 months) abscess or infection of the CNS, previous oncological disease other than nonmelanoma skin cancer and carcinoma in situ of the cervix, patients with severe psychiatric disease (eg, schizophrenia requiring recent hospitalization) and patients with developmental disorders (eg, infantile autism) or dementia. Pregnancy was also categorized as an exclusion criterion. Patients taking ≥48 mg methylprednisolone (≥16 mg TID) or ≥12 mg dexamethasone (≥4 mg TID) daily, were classified as receiving high-doses of steroids. Patients who received increasing doses of steroids up to the predefined time points for potential enrollment in a trial were also classified into a separate group. Polypharmacy was considered present if a patient was taking ≥5 drugs requiring a prescription from a physician (except for newly started drugs related to GBM).

### Ethics

The study was approved by The Norwegian Regional Committee for Medical Research Ethics (REK 2018/2295) and the data protection officers at the CRN and OUH (PVO 2017/7084).

### Statistical Considerations

We constructed a custom-made database using FileMaker Pro 16.0.5. Data analysis and graphic presentation were performed using GraphPad Prism 8.0, Microsoft Excel 16.3, and Keynote 10.2. Population characteristics are presented as observed counts, weighted percentages, and medians. Overall survival was calculated from the time of surgery to the time of death by the Kaplan–Meier method. In patients lacking tissue-based diagnosis survival was calculated from the time of diagnosis as defined by the CRN to the time of death. Differences between groups were compared by unpaired nonparametric Mann–Whitney *U* test. Survival between different groups and associations with overall survival were compared by the log-rank test. A *P*-value <.05 was considered significant.

## Results

### Population Characteristics

A total of 512 patients were diagnosed with GBM between 2012 and 2017, representing an incidence of 3.89 per 100 000/y. The diagnosis was made via tissue examination by a pathologist in 484 cases (94.5%), while 28 (5.5%) had their diagnosis derived from radiologic imaging only, as they were not considered candidates to undergo an invasive procedure. The median age of the entire population at diagnosis was 64 years, 133 patients (26%) were >70 years, and the median age of the patients with and without a tissue-based diagnosis was 63 and 81 years, respectively. There was a slight male predominance (1.53:1). Ten patients (2%) had disease involving both supra- and infratentorial regions, while 10 (2%) had solely infratentorial disease localized in the cerebellum (*n* = 5), brain stem (*n* = 3), or spinal cord (*n* = 2). A good performance status (ECOG **≤**2) at the time of diagnosis was observed in 469 patients (91.6%). Another previous or concurrent oncological disease was the most common comorbidity (*n* = 75, 14.6%). Further population characteristics are outlined in Table 1 and Supplementary Figure S1.

**Table 1.** Population Characteristics

	Number (%)	Median age (range) in years	Median survival (95% CI), months	HR (95% CI)	*P*-value
All patients	512 (100)	64 (10–97)	11.7 (10.8–12.8)	-	-
Basis of diagnosis					
Pathology	485 (94.5)	63 (10–89)	12.3 (11.4–13.2)	Reference	-
Radiology	28 (5.5)	81 (68–97)	2.4 (1.5–3.7)	6.54 (2.59–16.53)	<.0001
Gender					
Male	310 (60.5)	64 (10–97)	11.6 (10.8–12.8)	Reference	-
Female	202 (39.5)	64 (27–87)	11.9 (9.8–13.7)	0.87 (0.72–1.04)	.13
Localization					
Supratentorial	492 (96.0)	64 (11–97)	11.8 (11.0–12.9)	Reference	-
Supra- and infratentorial	10 (2.0)	49 (10–73)	7.4 (0.5–31.2)	1.13 (0.56–2.26)	.73
Infratentorial only	10 (2.0)	62 (17–69)	8.3 (1.3–40.2)	1.12 (0.56–2.24)	.74
Performance status, preoperatively					
ECOG 0	341 (66.6)	62 (10–83)	13.7 (12.4–14.8)	Reference	-
ECOG 1	99 (19.3)	66 (34–94)	9.3 (7.9–10.8)	1.79 (1.37–2.35)	<.0001
ECOG 2	29 (5.7)	67 (35–86)	7.0 (4.0–12.9)	2.02 (1.18–3.45)	.0003
ECOG 3	16 (3.1)	68 (45–84)	3.6 (1.5–12.4)	2.92 (1.27–6.69)	<.0001
ECOG 4	24 (4.7)	73 (11–97)	2.8 (1.1–6.0)	2.78 (1.39–5.54)	<.0001
N/A	3 (0.6)	85 (83–87)	2.0 (2.0–2.3)	-	-
Surgical procedure					
Resection	413 (80.7)	63 (10–84)	13.2 (12.4–14.1)	Reference	-
Open biopsy	33 (6.4)	69 (36–80)	8.4 (4.2–10.7)	2.33 (1.39–3.91)	<.0001
Stereotactic biopsy	38 (7.4)	65 (22–89)	3.7 (3.1–7.8)	3.23 (1.84–5.67)	<.0001
No surgery	28 (5.5)	81 (68–97)	2.4 (1.5–3.7)	8.03 (2.89–22.32)	<.0001
Age at diagnosis					
<40 y	31 (6.0)	32 (10–39)	31.2 (11.8–45.0)	Reference	-
40–49 y	51 (10)	46 (40–49)	15.4 (11.6–21.8)	1.45 (0.88–2.37)	.15
50–59 y	110 (21.5)	54 (50–59)	14.3 (12.9–17.0)	1.76 (1.19–2.61)	.012
60–69 y	172 (33.6)	65 (60–69)	12.5 (11.2–13.9)	2.34 (1.68–3.26)	<.0001
≥70 y	148 (28.9)	74 (70–97)	6.6 (4.8–8.4)	3.47 (2.53–4.75)	<.0001

Baseline characteristics of the patient population in the present study reported as observed counts and percentages, median age with range, median survival with 95% CI and hazard ratios of death.

ECOG, Eastern Cooperative Oncology Group.

### Patterns of Care and Survival in a GBM Population

**Figure 1.**
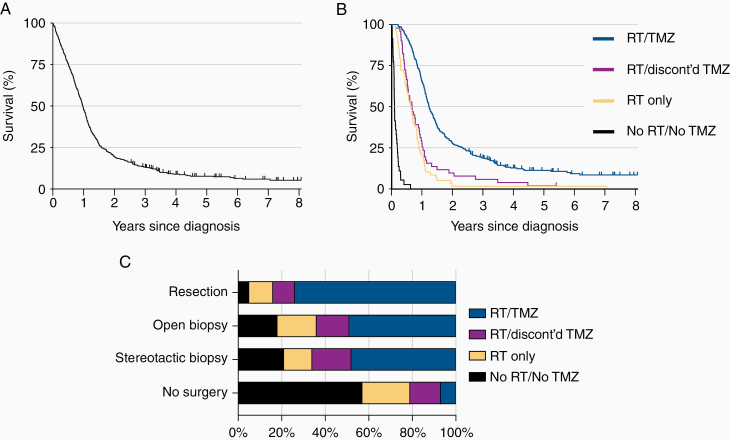
Patterns of care and survival in a GBM population. (A) Overall survival in the whole population. Vertical line represents censored events. (B) Overall survival of pathologically confirmed GBM stratified according to postoperative oncological treatment. (C) Receipt of RT and TMZ among the patients by type of surgery. GBM, glioblastoma; RT, radiotherapy; TMZ, temozolomide.

The median survival in the total population (*n* = 512) was 11.7 months, with 2- and 5-year survival rates of 19.3% and 7.4%, respectively (Figure 1A). The median survival times for patients with and without a tissue diagnosis were 12.3 and 2.4 months, respectively.

Among patients with a histological diagnosis (*n* = 484), 336 patients (69.4%) completed concurrent RT/TMZ, whereas 51 (10.5%) received RT (any dose) and started, but discontinued TMZ, 58 (12.0%) received RT only (any dose), and 36 (7.5%) received no RT or TMZ. In the group where GBM was confirmed by tissue analysis, the median survival was 14.8 months (95% CI 13.8–16.4) for patients who completed concurrent RT/TMZ, 8.0 months (6.2–10.9) for patients who received RT (any dose) and started, but discontinued TMZ, 7.6 months (5.7–9.7) for patients who received RT only (any dose), and 1.1 months (0.9–1.9) for patients who did not receive any additional oncological therapy (Figure 1B, *P* < .0001).

Of the patients who received TMZ in the concurrent phase, 1.5% (*n* = 6) developed leucopenia below the predefined threshold level of 1.5 × 10^9^/L corresponding to a grade 3 toxicity or higher, 8.3% (*n* = 33) developed thrombocytopenia below the predefined threshold level of 100 × 10^9^/L corresponding to a grade 2 toxicity or higher, and 2.3% (*n* = 9) elevated ALT, AST or bilirubin levels above the predefined levels corresponding to a grade 2 toxicity or higher, that required discontinuation of TMZ. Further treatment and survival characteristics are outlined in Figure 1C and Supplementary Table S2.

### Proportion of Patients Found Ineligible for a Phase III Trial

**Figure 2.**
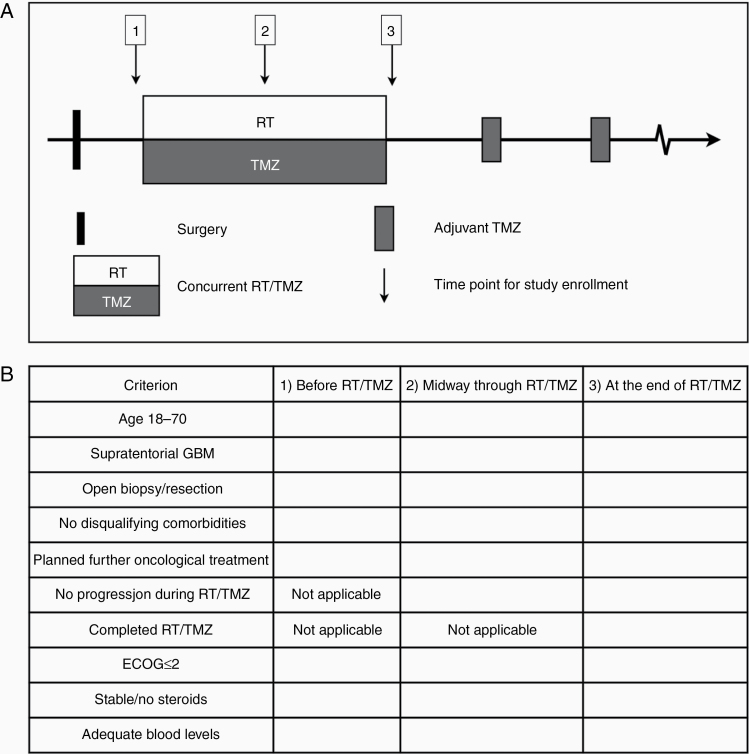
Time points and criteria for trial recruitment. (A) Potential time points for study enrollment during the concurrent RT/TMZ-phase (inset 1 to 3). (B) The inclusion and exclusion criteria a patient must fulfill to be considered trial participation in this study.

Phase III trials conducted in patients with newly diagnosed GBM over the last 10 years have enrolled patients at 3 different time points; (1) before the concurrent RT/TMZ (*n* = 5),^8,10,11,14,16^ (2) midway through RT/TMZ (*n* = 1),^9^ and (3) at the end/after completion of concurrent RT/TMZ (*n* = 4, Figure 2A).^7,12,13,15^ Although inclusion and exclusion criteria varied between individual trials, they broadly encompassed the same set of criteria (Supplementary Table S1). Commonly, these trials recruited patients within defined age limits (18–70 y), with a supratentorial located tumor, who underwent an open biopsy or surgical resection for tissue-based diagnosis and had a good performance status (ECOG **≤**2). On the other hand, they typically excluded patients with biochemical signs of hematologic, renal, or hepatic insufficiency, other predefined disqualifying comorbidities (eg, recent intracranial bleeding or infection, decompensated heart or lung disease, history of previous malignancy), disease progression before the time of randomization and patients taking increasing or high doses of steroids (Supplementary Table S1). Based on the inclusion and exclusion criteria that were used in the majority of the reference trials,^7–16^ we established a list of criteria a patient had to fulfill to be considered eligible for trial recruitment at 3 different time points (Figure 2B).

**Figure 3.**
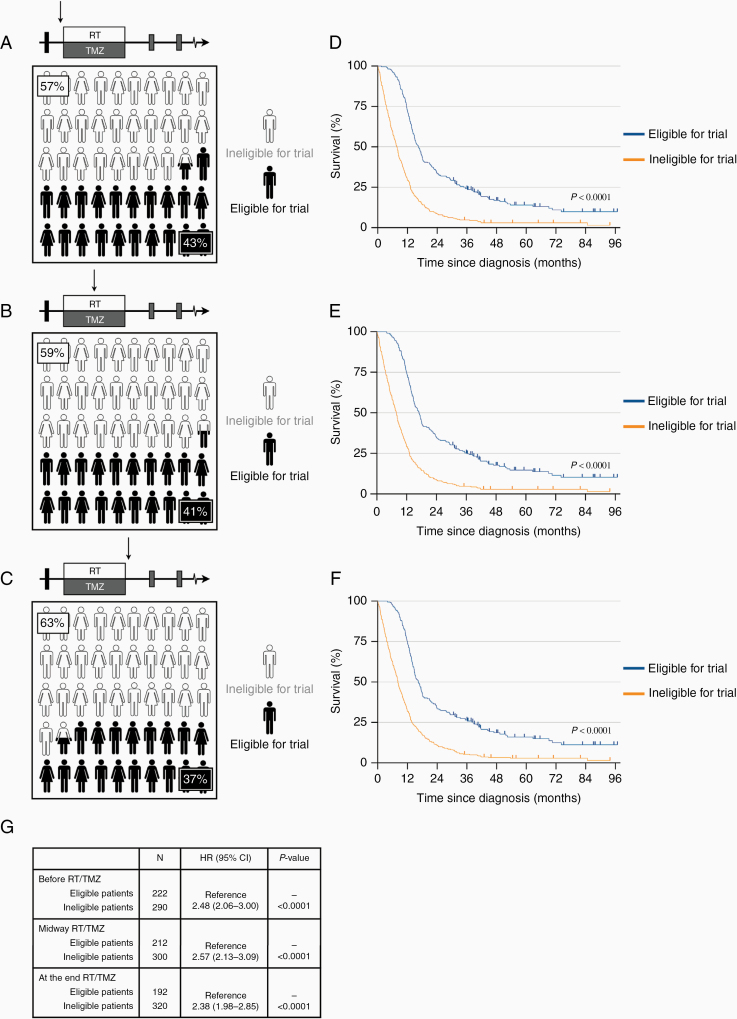
Simulated study group derived from a GBM population. (A) When filtering patients according to the selected study criteria, 57% of patients were considered ineligible for a clinical trial before the concurrent RT/TMZ. (B) Midway through RT/TMZ, the filtering excluded 59% of the patients, and (C) 63% of the patients were considered ineligible at the end of concurrent RT/TMZ. (D–F) Survival curves of patients found eligible compared to those found ineligible. There was a significant survival advantage (*P* < .0001) with over 2 times increased median, 2-y and 5-y survival at all time points for the patients found eligible for trials. (G) Hazard ratios of death of the patients found ineligible compared to those found eligible. RT, radiotherapy; TMZ, temozolomide.

Using these criteria, we simulated enrollment before planned concurrent RT/TMZ by filtering by age (18–70), localization (supratentorial), undergone open biopsy or surgical resection, no disqualifying comorbidity, good performance status (ECOG ≤2), stable doses, or no glucocorticoids, and adequate hematologic, hepatic and renal function. Patients were considered ineligible if they failed one or more of these criteria. This selection excluded 290 (57%) of the patients from a potential clinical trial (Figure 3A, Supplementary Table S3). We next simulated potential enrollment midway through concurrent RT/TMZ treatment by adding the criterion that patients had to have started concurrent RT/TMZ treatment without signs of disease progression. With this scenario, 300 (59%) patients were ineligible for trial participation (Figure 3B). When we finally filtered by the criterion that patients had to have completed concurrent RT/TMZ, that is, simulating inclusion following completion of the concurrent radiochemotherapy phase, 320 (63%) of the patients were excluded (Figure 3C).

### Survival Characteristics of the Potential Study Groups

We next compared the survival characteristics for eligible versus ineligible patients at different time points. Patients who potentially could have been included in a trial before the start of concurrent RT/TMZ had a median survival of 16.4 months, and the 2- and 5-year survival rates were 33.3% and 8.6%, respectively. Comparable data for the ineligible patients were 7.7 months, 14.0% and 3.0% (Figure 3D). Similar differences in survival were found at the two other time points for study inclusion (Figure 3E, F). The survival advantage for patients who fulfilled the eligibility criteria for trials at each time point was statistically significant (*P* < .0001, log-rank test). Compared to the group of patients who were considered ineligible for trials, the median, 2-year and 5-year survival rates were increased >2 times in the group of patients considered trial candidates (Figure 3D–F, Table 2).

**Table 2.** Characteristics of Patients Considered Trial Eligible

	Before RT/TMZ		Midway RT/TMZ		At the end of RT/TMZ	
	Eligible	Ineligible	Eligible	Ineligible	Eligible	Ineligible
Median age, years	58.0	69.0	58.0	68.5	58.0	68.0
Male:female ratio	1.7:1	1.4:1	1.6:1	1.5:1	1.9:1	1.4:1
ECOG, median	0	1	0	1	0	1
Surgical resection, %	98	67	98	68	98	70
Median survival, months	16.4	7.7	17.0	7.9	16.4	8.2
2-y survival, %	33.3	14.0	34.4	14.7	33.3	16.0
5-y survival, %	8.6	3.0	8.7	2.9	10.9	2.9

Characteristics of GBM patients considered to be trial eligible compared to those found ineligible for trials at different time points.

ECOG, Eastern Cooperative Oncology Group; GBM, glioblastoma.

### Patient Characteristics of the Potential Study Groups

We further compared the characteristics of patients who were considered eligible for a trial to those considered ineligible. Before the start of concurrent RT/TMZ, compared to median age of 69.0 years for those found ineligible, the median age of patients who fulfilled the criteria for study enrollment was 58.0 years (*P* < .0001, Mann–Whitney test). Similar differences were found at both of the other time points for study enrollment (both *P* < .0001). In the group of patients considered eligible for trial recruitment, compared to a median ECOG of 1 among the ineligible patients, the median ECOG performance status was 0. Compared to 67% among ineligible patients, a total of 98% of patients considered trial candidates had undergone tumor resection surgery. The characteristics between the groups are outlined in Table 2.

We used data from the reference trials for external validation.^7–16^ Except for the survival rate, which was extracted from the control group of each trial, we utilized data from both randomized cohorts in the trials. We left out survival data from 2 trials^8,16^ that exclusively recruited MGMT-methylated tumors (as they have significantly better survival), and from one trial with no control group.^15^ We found that our data on patients (age, male to female ratio, ECOG status, a proportion that underwent surgical resection, median- and 2-year survival) considered to be candidates for trial recruitment corresponded well with the patients that *de facto* had been participants in clinical trials for GBM (Table 3).

**Table 3.** Patient Characteristics of Trial Participants

Reference phase 3 trials in GBM (*n* = 10)			
Trial Participants		No. of Trials	References
Median age of randomized cohorts, years (range)	56.5 (54.0–59.0)	9^a^	^8–16^
Male:female ratio (range)	1.5:1 (1.2–2.1:1)	10	^7–16^
ECOG, median	0-1^b^	10	^7–16^
Surgical resection, median % (range)	97 (87–100)	10	^7–16^
Median survival, months (range)	16.8 (16.0–20.0)	7^c^	^7–9,11–14^
2-year survival, median % (range)	33.9 (30.1–40.0)	5^d^	^7,9,11–13^

Characteristics of trial participants extracted from the phase 3 trials used as reference trials.

ECOG, Eastern Cooperative Oncology Group.

^a^One study did not report median age of the patients.

^b^Performance status is reported over an interval as it was converted from KPS in 8 studies that could not discern a true median value.

^c^Three studies were excluded from survival calculations as 2 recruited MGMT-methylated tumors and one did not have an adequate control group.

^d^Only 5 studies specifically reported 2-year survival characteristics.

## Discussion

Our study provides a quantitative portrait of the patients who can be considered either eligible or ineligible for enrollment into phase III trials for newly diagnosed GBM. Our main result is that only a minority of the patients are eligible for inclusion into a typical RCT. Compared to a real-world GBM population, this minority formed a more homogeneous group consisting of younger patients with a better performance status that were more likely to have undergone tumor resection surgery. As these factors are associated with improved survival,^17–19^ it was not surprising that compared to those considered ineligible for trial participation, survival rates were markedly improved.

Our conclusion depends on; (1) that the study population represents a true real-world GBM population with patterns of care corresponding to that seen in recent population-based series, and (2) that our eligibility criteria result in the selection of a group of patients who has the characteristics of patients included in recent RCTs.

All patients within our defined geographical area with an intracranial lesion suspicious of GBM were referred to one hospital (OUH). Irrespective of how the diagnosis of GBM is made and the treatment modalities are applied, all patients are by law reported to the national CRN, where the diagnosis and further details of the disease are stored with the patient’s home address and unique national ID. The risk for missing individual patients in the current study was therefore small. We found an annual incidence of 3.89/100 000. The median age at diagnosis was 64 years, 26% of patients were over 70 years old and there was a slight male predominance. In addition, 2% presented with the infratentorial disease. These data are in accordance with several epidemiological series over time and across different GBM populations,^22–29^ indicating that our population corresponds well to established nonadjustable epidemiologic features in GBM.

We further found that the diagnosis was made without tissue examination (ie, by MRI only) in 5.5% of the cases, compared to around 5–20% in previous reports.^2–4,30–32^ Among the patients with a tissue diagnosis, resection surgery was performed in 80%, and 2/3 completed concurrent RT/TMZ. These levels correspond to patterns of care in other population-based series (70–90% of patients undergoing resection surgery,^18,33,34^ and approximately 60% receiving first-line treatment of concurrent RT/TMZ^2,3,34,35^), as do our data on overall, 2- and 5-year survival rates.^4,5,26,35^ We, thus, conclude that also the patterns of care and survival characteristics in this study are comparable to those in other recent population-based series.

When comparing the characteristics of the cohorts of patients who were included in the reference trials to the group found eligible for a typical RCT in this study, we found the median age to be 56.5 years (range 54.0–59.0) in the reference trials, and 58.0 years in the patients we found trial eligible. Compared to a median for 97% (range: 87–100%) in the reference trials, resection surgery was performed in 98% of patients considered trial candidates. Before comparing performance status before treatment, we converted performance data from all studies to the same scale (ECOG).^36^ Compared to a median of 0–1 in the reference trials (as several trials reported their baseline data over a range, we could not discern the true median other than over a range), the median ECOG among the patients we found eligible was 0. Lastly, depending on the different time points the median overall survival of patients we found eligible for a potential RCT ranged from 16.4–17.0 months versus 16.8 (range: 16.0–20.0) months in the reference trials. Correspondingly, the median 2-year survival ranged from 33.3–34.4% in our data versus 33.9% (30.1–40.0%) in the reference trials. Collectively, our data, therefore, indicate that the selection criteria we applied in the current study resulted in a group representative for patients typically included in a phase III trial in GBM. This substantiates our main conclusion that only a minority of patients with a newly diagnosed GBM is eligible for inclusion into a typical RCT.

The eligibility criteria applied in the reference trials were largely equivalent with respect to performance status, mode of diagnosis, physiological parameters, and disqualifying comorbidities.^7–16^ Although only 4 studies used 70 years as an upper age limit, enrolled patients tended to be younger also in the investigation where no such restriction was imposed.^9,10,13^ In line with the Stupp trial,^37^ we chose to use 70 years as an upper limit for potential trial inclusion. Still, we ended up with 58.0 years as the median age for trial eligible patients, compared to median age of the randomized patients ranging from 54.0 to 59.0 in the reference trials.^7–16^ As the median age at diagnosis in our unselected population was 64 years, we suspect that there has been a tendency to exclude older patients even in the studies where the age limit was not applied.

Phase III clinical trials are fundamental for establishing standards of care. Our data, where approximately 60% of patients are excluded from trials, suggest that current trial protocols only provide treatment guidance to a minority of the patients. As the generalizability of trial results is restricted to the population that has been studied, it remains uncertain how the majority should be treated. Unfortunately, the patients with the worst prognosis, where new treatment options are needed the most, are represented in this group.

It is well known that patients in clinical trials may be selected to such an extent that study results do not directly apply to the real-world situation.^6,38^ In brain tumors, skewed inclusion was described for anaplastic glioma by Macdonald and Cairncross’ group over 3 decades ago.^39^ Later studies evaluating survival of glioma patients described significant survival advantage of those considered eligible for experimental trials.^40,41^ In accordance with our data, these studies described the systematic skewness of younger patients with better performance status undergoing more extensive surgical treatment in the population considered trial eligible. Despite standards developed for brain tumor therapy trials,^42,43^ it remains a problem that only a minority of patients are included in such trials.

The major strengths of this study lie in the unique surveillance of GBM within a well-defined geographical area enabled by the CRN and BTD, as well as in the number of patients, the individual-level data, and that the results were derived from a real-world population setting. Moreover, the data were restricted to one health center only (OUH), thereby reducing the possible confounding effect of differences in access to health care services between health centers.

The major limitation of the study is the retrospective sampling of some of the data from individual patient records. As this introduces a degree of subjectivity primarily affecting scoring of ECOG status and patient comorbidity, 2 independent researchers went through the data of each patient. The retrospective design, moreover, cannot discern certain variables that might affect trial eligibility, such as logistical issues as well as attitudes and willingness to participate in a trial, that further exclude patients from RCTs.^7–16,38^ A further limitation is the lack of molecular data, especially IDH- and MGMT methylation status. This aspect is important as the stratification of patients according to molecular data will be important in future trials.

Concerns have been raised with regard to the low accrual rate of patients into neuro-oncological trials, which is estimated to be only approximately 10%.^44^ Barriers for trial accrual are related both to physicians (eg, awareness), and organizations/institutions (eg, infrastructure, economy).^45^ In addition, adjustments of eligibility criteria for trials to increase enrollment have gained increasing attention in the academic community in recent years.^46,47^ In this study, most patients were excluded based on age and comorbidities. With an increasingly aging population, it is expected that the number of patients with GBM will increase.^5^ To enroll more patients in trial accepting a wider age span, as well as allowing patients with a history of the malignant disease whose natural history or treatment does not have the potential to interfere with trial endpoints, may therefore be the two most useful criteria to increase the fraction of eligible patients.

In summary, we have provided a quantitative portrait of the selection bias of GBM patients in phase III clinical trials. We have shown that only a minority of patients in a real-world GBM population can be expected to fulfill standard eligibility in such trials and that these patients are younger, have better performance status and fewer comorbidities, and are more likely to have undergone tumor resection. Consequently, cohorts included in RCTs have improved survival compared to an unselected real-world population. This selection bias should be taken into consideration when trial results are used to guide treatment.

## Supplementary Material

vdab008_suppl_Supplementary_Figures_and_Tables

vdab008_suppl_Supplementary_Legends

## Notes

**Authorship Statement:** Conceived the study and study design: E.S., P.B., E.O.V.M., E.H., and I.A.L. Collected data: E.S., M.A.S., T.B.J., E.H., and I.A.L. Assisted in analyses: E.S., M.A.S, and I.A.L. Interpreted the data: E.S., T.B.J., P.B., E.O.V.M., E.H., and I.A.L. Wrote the manuscript: E.S., E.O.V.M., and I.A.L. All authors have seen and approved the final manuscript and submission.
