# Supplementary material for: Real-world validity of randomized controlled phase III trials in newly diagnosed glioblastoma: to whom do the results of the trials apply?
Source: Neurooncol Adv. 2021 Feb 26;3(1):vdab008. doi: 10.1093/noajnl/vdab008 (PMC7914075; doi:10.1093/noajnl/vdab008)
Supplement: vdab008_suppl_Supplementary_Figures_and_Tables [file vdab008_suppl_supplementary_figures_and_tables.pdf]

Supplementary Table S1

Criteria for study enrollment		Gilbert JCO (2013)	Gilbert NEJM (2014)	Chinot NEJM (2014)	Stupp Lancet (2014)	Westphal EJC (2015)	Stupp JAMA (2017)	Kong Oncotarget (2017)	Weller Lancet Oncol. (2017)	Liau JTM (2018)	Herrlinger Lancet (2019)
Age	18-70				§	●		●	§	●	●
	≥ 18	●	●	●	●		●		●		
Performance status	ECOG 0-2<br>*ECOG 0-1 only			●	* ●				●		
	KPS ≥ 70<br>*KPS ≥ 60	* ●	●	●		●	●	* ●		●	●
Physiology	Adequate hematologic, hepatic & renal function<br>*Adequate coagulation	●	●	* ●	●	●	●	* ●	●	●	* ●
Pathology	Newly diagnosed glioblastoma<br>*Specify supratentorial	* ●	●	* ●	* ●	●	* ●	●	* ●	* ●	●
Tretment	Bopsy/resection<br>*Resection required	●	* ●	●	●	●	●	●	* ●	* ●	●
	RT/TMZ<br>#Planned for 60Gy/TMZ<br>*Completed RT/TMZ	* ●	# ●	# ●	# ●	# ●	* ●	# ●	* ●	* ●	# ●
Glucocorticoids	Stable or decreasing doses	●	●	●	●				●		●
Study specific criteria for enrollment	E.g. molecular stratification				MGMT methylated GBMs only				EGFRvIII positive GBMs only		MGMT methylated GBMs only
Disease status	Progressive disease before randomization	●	●	●	●		●		●	●	
Disqualifying comorbidities	E.g. active heart, lung, hepatic, renal disease, other malignancy etc.	●	●	●	●	Not described	●	●	●	●	●

	Gilbert JCO (2013)	Gilbert NEJM (2014)	Chinot NEJM (2014)	Stupp Lancet (2014)	Westphal EJC (2015)	Stupp JAMA (2017)	Kong Oncotarget (2017)	Weller Lancet Oncol. (2017)	Liau JTM (2018)	Herrlinger Lancet (2019)
Time of randomization	**After** completion of concurrent RT/TMZ	Randomization after **3 weeks (midway) into RT/TMZ**	**Before** start of concurrent RT/TMZ	**Before** start of concurrent RT/TMZ	**Before** start of concurrent RT/TMZ	**After** completion of concurrent RT/TMZ	**Before** start of concurrent RT/TMZ	**After** completion of concurrent RT/TMZ	**After** completion of concurrent RT/TMZ	**Before** start of concurrent RT/TMZ

Inclusion criteria for enrollment trials

Inclusion criteria used in a minority of trials investigating specific molecular subgroup (not considered a criteria in this study)

Exclusion criteria for enrollment in trials

§ The trials do not report an upper age limit as an inclusion criterion, however, the patient characteristics do not descibe any patients above 70 years (the reported median age and ranges < 70 years), and they do not describe any data for patients > 70 years in the article nor supplementary material.

Supplementary Table S2

	No RT/no TMZ		RT (any dose)/no TMZ		RT (any dose) /discontinued TMZ		Completed RT/TMZ		Total (%)
	Number (%)	Median survival, months	Number (%)	Median survival, months	Number (%)	Median survival, months	Number (%)	Median survival, months	
No surgery	16 (3.1)	1.7	6 (1.1)	3.4	4 (0.8)	6.4	2 (0.4)	8.6	28 (5.5)
Stereotactic biopsy	8 (1.6)	1.6	5 (1.0)	2.7	7 (1.4)	5.5	18 (3.5)	8.5	38 (7.4)
Open biopsy	6 (1.2)	0.9	6 (1.2)	6.0	5 (1.0)	6.5	16 (3.5)	10.7	33 (6.4)
Resection	22 (4.3)	1.1	47 (9.2)	8.2	39 (7.6)	9.3	302 (59.0)	15.6	410 (80.1)
Total (%)	52 (10.2)	1.3	64 (12.5)	6.7	55 (10.7)	8.0	338 (66.0)	14.8	509* (99.4%)[a]

Supplementary Table S3

Criterion	1) Before RT/TMZ (n=excluded)	2) Midway through RT/TMZ (n=excluded)	3) At the end of RT/TMZ (n=excluded)
Age 18-70	136	136	136
Supratentorial GBM	17	17	17
Open biopsy/resection	29	29	29
No disqualifying comorbidities	72	72	72
Planned further oncological treatment	23	23	23
No progressjon during RT/TMZ	Not applicable	4	5
Completed RT/TMZ	Not applicable	Not applicable	12
ECOG ≤2	5	6	3
Stable/no steroids	8	11	6
Adequate blood levels	0	2	17
Total excluded patients (%)	290 (57%)	300 (59%)	320 (63%)

Supplementary Figure S1

A

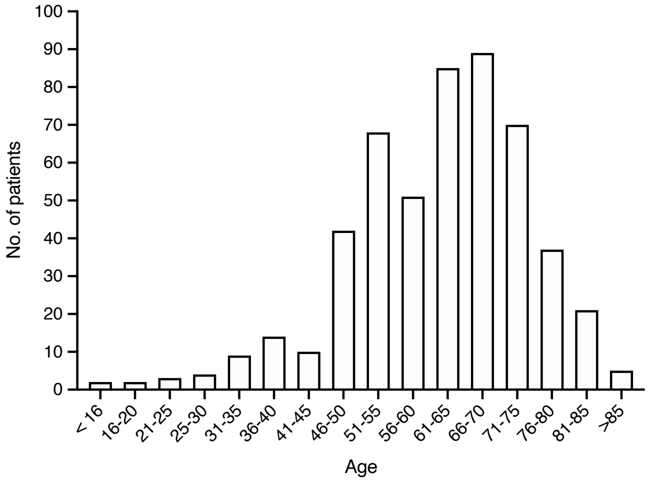

B

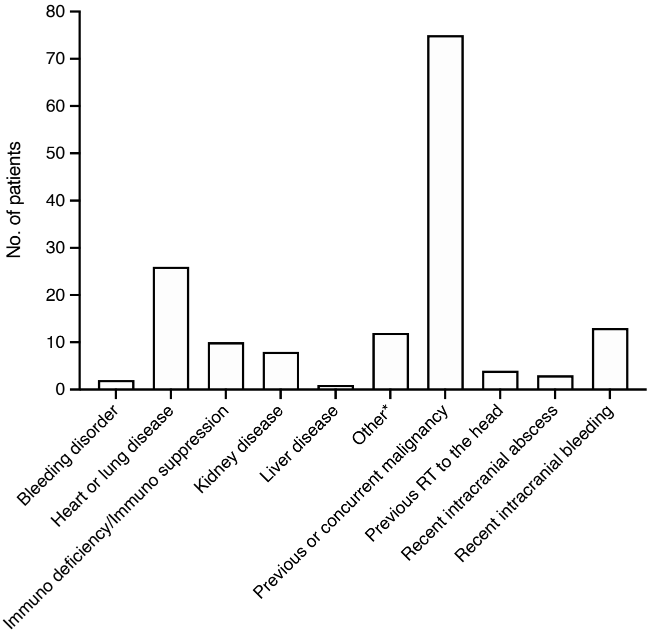

*Diagnoses within the category: dementia, congenital intellectual disability, severe psychiatric disorder, downs syndrome. Also one patient that was pregnant.

C

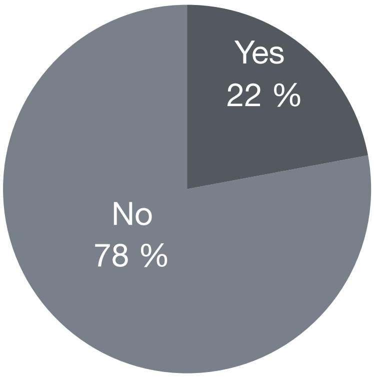

	Number (%)	Median age (range) in years	Median survival (95% CI) in months
Polypharmacy			
Yes	113 (22.0)	69 (37-85)	10.1 (8.1-11.9)
No	399 (78.0)	61 (10-97)	12.1 (11.1-13.3)
