# Supplementary material for: Real-world validity of randomized controlled phase III trials in newly diagnosed glioblastoma: to whom do the results of the trials apply?
Source: Neurooncol Adv. 2021 Feb 26;3(1):vdab008. doi: 10.1093/noajnl/vdab008 (PMC7914075; doi:10.1093/noajnl/vdab008)
Supplement: vdab008_suppl_Supplementary_Legends [file vdab008_suppl_supplementary_legends.docx]

**Supplementary table legends**

***Supplementary Table S1. Phase 3 trials conducted in GBM used as reference trials in this study.*** *Overview of the inclusion and exclusion criteria used in the clinical trials used as reference trials for this study.*

***Supplementary Table S2. Patterns of care and survival characteristics.*** *Overview summarizing observed counts and percentages with median survival stratified by surgical and adjuvant oncological treatment. ^a^Three patients that only received only TMZ (no RT) are not included in the table.*

***Supplementary Table S3.Patient ineligible for clinical trials.*** *List and number of excluded patients according to inclusion and exclusion criteria applied in this study.*

**Supplementary figure legends**

***Supplementary Figure S1. Additional population characteristics.*** *Age-distribution, frequency of comorbidities and patients defined with polypharmacy in the population.*
